# Interplay Between EGFR and the Platelet-Activating Factor/PAF Receptor Signaling Axis Mediates Aggressive Behavior of Cervical Cancer

**DOI:** 10.3389/fonc.2020.557280

**Published:** 2020-12-17

**Authors:** Juliana L. Souza, Karina Martins-Cardoso, Isabella S. Guimarães, Andréia C. de Melo, Angela H. Lopes, Robson Q. Monteiro, Vitor H. Almeida

**Affiliations:** ^1^ Instituto de Bioquímica Médica Leopoldo de Meis, Universidade Federal do Rio de Janeiro, Rio de Janeiro, Brazil; ^2^ Divisão de Pesquisa Clínica e Desenvolvimento Tecnológico, Instituto Nacional de Câncer, Rio de Janeiro, Brazil; ^3^ Instituto de Microbiologia Paulo de Góes, Universidade Federal do Rio de Janeiro, Rio de Janeiro, Brazil

**Keywords:** cervical cancer, epidermal growth factor receptor, platelet-activating factor, platelet-activating factor receptor, lysophosphatidylcholine acyltransferase 2, signaling pathways

## Abstract

Epidermal growth factor receptor (EGFR) is a receptor tyrosine kinase widely expressed in cervical tumors, being correlated with adverse clinical outcomes. EGFR may be activated by a diversity of mechanisms, including transactivation by G-protein coupled receptors (GPCRs). Studies have also shown that platelet-activating factor (PAF), a pro-inflammatory phospholipid mediator, plays an important role in the cancer progression either by modulating the cancer cells or the tumor microenvironment. Most of the PAF effects seem to be mediated by the interaction with its receptor (PAFR), a member of the GPCRs family. PAFR- and EGFR-evoked signaling pathways contribute to tumor biology; however, the interplay between them remains uninvestigated in cervical cancer. In this study, we employed The Cancer Genome Atlas (TCGA) and cancer cell lines to evaluate possible cooperation between EGFR, PAFR, and lysophosphatidylcholine acyltransferases (LPCATs), enzymes involved in the PAF biosynthesis, in the context of cervical cancer. It was observed a strong positive correlation between the expression of EGFR × PAFR and EGFR × LPCAT2 in 306 cervical cancer samples. The increased expression of LPCAT2 was significantly correlated with poor overall survival. Activation of EGFR upregulated the expression of PAFR and LPCAT2 in a MAPK-dependent fashion. At the same time, PAF showed the ability to transactivate EGFR leading to ERK/MAPK activation, cyclooxygenase-2 (COX-2) induction, and cell migration. The positive crosstalk between the PAF-PAFR axis and EGFR demonstrates a relevant linkage between inflammatory and growth factor signaling in cervical cancer cells. Finally, combined PAFR and EGFR targeting treatment impaired clonogenic capacity and viability of aggressive cervical cancer cells more strongly than each treatment separately. Collectively, we proposed that EGFR, LPCAT2, and PAFR emerge as novel targets for cervical cancer therapy.

## Introduction

Cervical cancer is the fourth most common tumor and the fourth leading cause of cancer death in women worldwide, with 570,000 cases and 311,000 deaths in 2018. Most cases of this neoplasia occur in low- and middle-income countries and the human papillomavirus (HPV) infection is the virtually necessary factor for cervical cancer development (1). Patients with advanced-stage tumors have 5-year survival rates lower than 50% (2), and novel therapeutic strategies are needed to improve these women’s prognosis.

Membrane proteins, such as receptor tyrosine kinases (RTKs) and G protein-coupled receptors (GPCRs), are critical in the intercellular communication and signal transduction, modulating gene expression and cell responses to the extracellular stimuli. Alterations in these receptors and their regulated signaling pathways are commonly observed in cell transformation and tumor progression, making them useful as biomarkers, prognostic factors, and pharmacological targets (3).

The human epidermal growth factor receptor (EGFR/HER-1) belongs to the HER (Human EGF Receptor) receptor tyrosine kinase family. EGFR activation by ligands leads to its dimerization, phosphorylation, and activation of signaling pathways, such as PI3K (phosphoinositide 3-kinase) - Akt, MAPK (mitogen-activated protein kinase)/ERK (extracellular signal-regulated kinase), STATs (signal transducer and activator of transcription), and PLC (phospholipase C) - PKC (protein kinase C), which promote cell proliferation, angiogenesis, apoptosis evasion and cell invasion (4, 5). The literature has shown that EGFR is expressed in about 80% of cervical carcinomas and is correlated with disease progression (6–8). In addition, previous reports demonstrated that EGFR can be transactivated by various agonists unrelated to EGFR ligands, such as GPCR ligands, in models that include cervical cancer cells (9–11).

Chronic inflammatory microenvironment has been suggested as an enabling characteristic of cell transformation and oncogenesis (12). Platelet-activating factor (PAF, 1-O-alkyl-2-acetyl-sn-glycero-3-phosphocholine) is a biologically active phospholipid mediator with potent pro-inflammatory activity. Although PAF has been named for its ability to induce platelet activation at nanomolar concentrations, several studies have revealed other PAF’s biological actions in a wide variety of cell types and tissues (13).

Functionally, there are two pathways for PAF biosynthesis: the *de novo* pathway and the remodeling pathway. The remodeling route is triggered by inflammation and is the main source of PAF under pathological situations. The initiation of the remodeling pathway requires hydrolysis of phosphatidylcholine (PC) by phospholipase A2 (PLA2), which generates a free fatty acid, such as arachidonic acid, and lyso-PC, a precursor of PAF. Lyso-PC acetyltransferases (LPCATs) then convert lyso-PC into PAF through acetylation in the sn-2 position. Finally, PAF activates the PAF receptor (PAFR), which belongs to the superfamily of GPCRs (14, 15). Inappropriate activation of the PAF-PAFR axis is thought to play an important role in cancer biology, tumor radioresistance, and modulation of the tumor microenvironment (16, 17).

Some studies have demonstrated the participation of EGFR-evoked signaling pathways as positive modulators of two key enzymes, cytosolic PLA2 (18) and LPCAT2 (15), involved in the remodeling route of PAF biosynthesis. Indeed, EGFR activation increases PAF production in ovarian cancer cell lines in a PLA2-dependent mechanism (19). PAF also transactivates EGFR and downstream pathways in ovarian cancer cells, diversifying the GPCR-mediated signal (20, 21). However, the role of the crosstalk between EGFR and the PAF-PAFR axis in other types of cancer has yet to be investigated.

**Figure 1:**
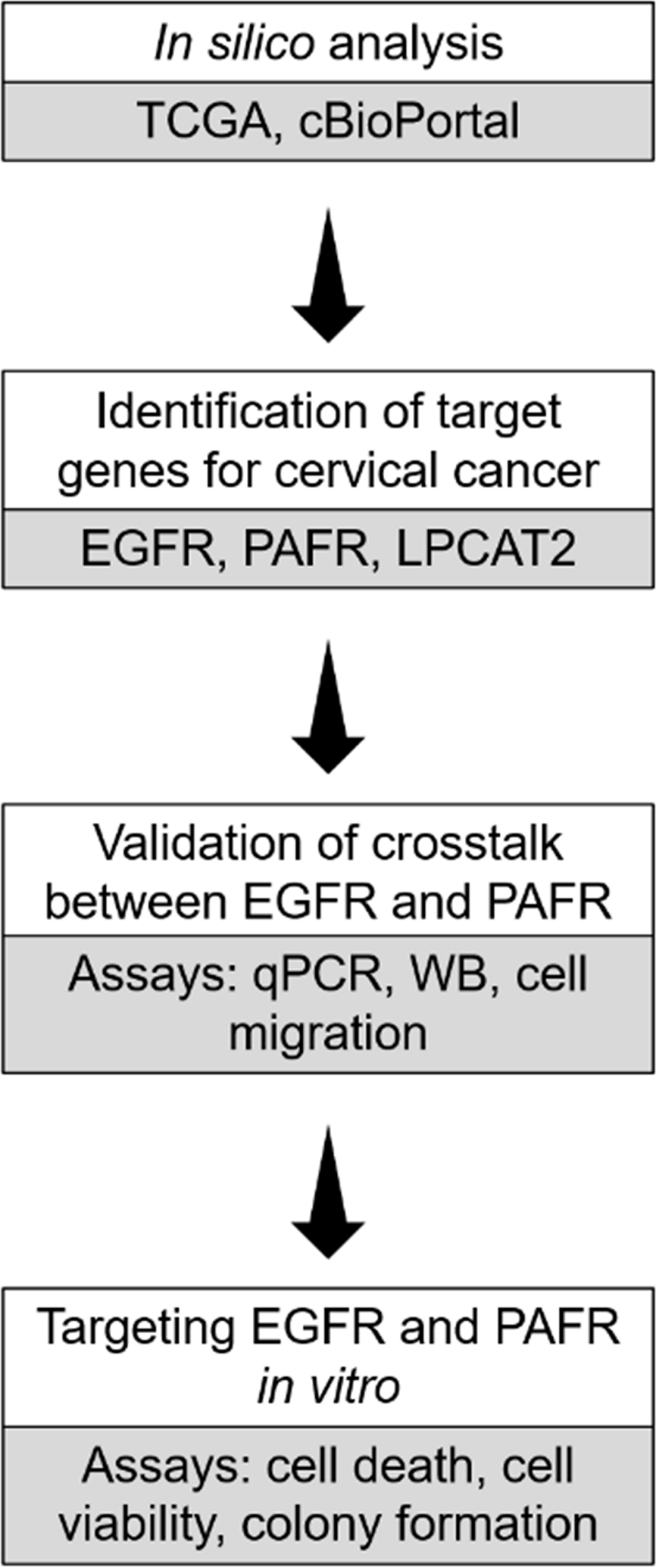
Experimental design of the study. *In silico* analysis were employed to identify genes that could potentially serve as targets in cervical cancer. *In vitro* assays were further utilized to validate the crosstalk between epidermal growth factor receptor (EGFR) and platelet-activating factor receptor (PAFR). In addition, EGFR and PAFR were tested as targets using *in vitro* assays.

Herein, we identified EGFR, LPCAT2, and PAFR as targets for cervical cancer therapy, using TCGA-based *in silico* analyzes. The bidirectional interaction between EGFR signaling pathway and the LPCAT-PAF-PAFR axis, and the functional impact of inhibiting both pathways with target drugs were investigated *in vitro*. The experimental design of the study is shown in **Figure 1**.

## Materials and Methods

### Correlation Analysis of Gene Expression

Transcriptome data were collected from The Cancer Genome Atlas (TCGA^1^). The subset of TCGA data included the RNA sequencing of 306 samples of cervical squamous cell carcinoma and cervical adenocarcinoma. RNA-seq values (*EGFR* × *LPCAT1*/*2*/*3*/*4*, *EGFR* × *PTAFR*, and *EGFR* × *PLA2G4A*) were correlated and statistically analyzed by the non-parametric Spearman test.

### Overall Survival Study

Overall survival analyzes were performed using the open-access platform named cBioPortal for Cancer Genomics (22, 23). To observe the relationship between the upregulation of PAFR (*PTAFR* gene), cPLA2 (*PLA2G4A* gene*)* and the four enzymes of the LPCAT family (*LPCAT1*/*2*/*3*/*4*) with the overall survival of cervical cancer patients, the Firehose Legacy study (TCGA, n = 304) was selected. A cut-off of 2.0 standard deviations above the median of expression (z-score) was established to separate the groups: “upregulated expression” and “cases without alteration”.

### Cell Lines and Chemicals

CASKI and C33A cells were grown in RPMI 1640 medium (Thermo Fisher Scientific, MA, USA) supplemented with 10% fetal bovine serum (FBS), 1% penicillin/streptomycin (Thermo Fisher Scientific), and incubated at 37°C in a 5% CO_2_ atmosphere. Unless otherwise stated, cell lines were seeded at 1 × 10^6^ cells in 25 cm^2^ culture flasks and left to adhere overnight.

Cetuximab (anti-EGFR monoclonal antibody, Erbitux^®^) was provided by the Brazilian National Cancer Institute (Rio de Janeiro, Brazil). WEB2086 (PAFR competitive antagonist), β-Acetyl-γ-O-alkyl-L-α-phosphatidylcholine (PAF), PD98059 (MEK inhibitor), LY294002 (PI3K inhibitor) and human epidermal growth factor (EGF) were purchased from Merck, Germany.

### Gene Expression Analysis by Quantitative PCR

After 16 h of starving in serum-free medium, cells were treated with cetuximab (100 μg/ml) or PD98059 (50 μM) or LY294002 (25 μM) for 90 min, when indicated. After treatment, cells were stimulated with PAF (100 nM) or EGF (50 ng/ml or 100 ng/ml) for 90 min. TRIzol Reagent was used to extract total RNA. From each sample generated, 1 μg of RNA was submitted to DNase I treatment and RT-PCR. Next, real-time PCR was performed on cDNA aliquots with Taqman Fast Real-Time PCR Master Mix, using the StepOnePlus Real-Time PCR System (Thermo Fisher Scientific). References regarding Taqman gene expression assays were 4326317E (*GAPDH*), Hs00153133_m1 (COX-2, *PTGS2* gene), Hs00265399_s1 (PAFR, *PTAFR* gene) and Hs01044164_m1 (*LPCAT2*). All reagents and probes were purchased from Thermo Fisher Scientific. Gene expression was normalized by *GAPDH* as a reference gene. To analyze the relative fold change, we employed the 2^−ΔΔCT^ method.

### Western Blotting

In experiments analyzing the signaling pathways, cells were starved for 16h and treated with cetuximab (100 μg/ml) for 90 min, when indicated. Afterward, cells were stimulated with PAF (100 nM) for 5 to 20 min. In experiments to evaluate COX-2 protein expression, the same protocol was used. However, treatment with PAF lasted 3 h. In experiments analyzing PAFR downregulation, CASKI cells were simultaneously starved and treated with cetuximab (100 µg/ml) for 24 h and 48 h. Following treatment completion, cells were lysed, and proteins were quantified using the Lowry method (DC protein assay, Bio-Rad, CA, USA). Protein lysates (20–30 μg) from each condition were subjected to 8%–10% SDS–PAGE and transferred onto a PVDF Hybond-P membrane (GE Healthcare, Brazil). Membranes were blocked and incubated overnight with p-ERK1/2 (Cell Signaling Technology, MA, USA), ERK1/2 (Cell Signaling Technology), COX-2 (Cell Signaling Technology, MA, USA), PAFR (Abcam, MA, USA) and β-actin (Cell Signaling Technology) primary antibodies. Subsequently, membranes were incubated with HRP-conjugated secondary antibodies (DakoCytomation, Denmark) for 1 h at room temperature, and immunoblots were detected using the ECL reagent (GE Healthcare, Brazil).

### Cell Migration Assay

The cell migration assay was performed in the Boyden chamber using 8 μm pore polycarbonate membranes (Neuro Probe, MD, USA). CASKI cells were seeded at 4 × 10^5^ cells per well in 6-well cell culture plates and allowed to adhere overnight. On the next day, after 10 h of starving in serum-free medium, cells were treated with cetuximab (100 μg/ml) for 90 min and then stimulated with PAF (100 nM) for approximately 16 h. After that, cells were washed and harvested in serum-free medium. To the upper chambers, 5 × 10^4^ cells were added, while the lower chambers were filled with RPMI containing 5% FBS to stimulate migration. After 20 h of incubation, non-migrated cells on the upper membrane surface were scraped, and the membranes were fixed and stained using fast staining (Panoptic, Laborclin, Brazil). Finally, the membrane was photographed at 100x magnification. Ten fields were counted, and each sample was performed in triplicate.

### Flow Cytometry-Based Cell Death Detection

CASKI cell line was seeded at 4 × 10^5^ cells per well in 6-well cell culture plates and allowed to adhere overnight. Next, cells were treated with cetuximab (100 μg/ml) and/or WEB 2086 (50 μM) for 24 h. After treatment, the supernatant was collected, and adherent cells were trypsinized. The material collected was centrifuged and washed twice with cold PBS (phosphate-buffered saline). Nicoletti buffer (0.1% sodium citrate, 0.1% NP-40, 200 μg/ml RNase and 50 μg/ml propidium iodide) was used to stain the DNA of the cells. Analysis of the DNA content was observed by collecting 20,000 events using the BD FACSCalibur flow cytometer (BD Biosciences, EUA). Doublets and debris were identified and excluded. Events with DNA hypodiploid (sub-G0/G1 peak) were considered as dead cells.

### Cell Viability Assay

CASKI was seeded at a density of 2,500 cells/well in 96-well cell culture plates. After overnight incubation, cells were treated with cetuximab (100 μg/ml) and/or WEB 2086 (100 μM) for 72 h in medium supplemented with 2% FBS. After treatment, cells were incubated with a solution of MTT (3-(4,5-dimethythiazol-2-yl)-2,5-diphenyl-tetrazolium bromide) (Merck, Germany) for 3.5 h. The formazan crystals formed were solubilized in DMSO, and the optical density was measured at a wavelength of 538 nm using Spectra Max ABS Plus (Molecular Devices, CA, EUA). Cell viability was expressed as a percentage of the control.

### Colony Formation Assay

CASKI cells were seeded at a low density of 300 cells per well in 6-well cell culture plates. On the next day, cells were treated with cetuximab (100 μg/ml) and/or WEB 2086 (50 μM) and allowed to grow for 13 days. After the incubation time, colonies were stained with 0.1% crystal violet (Merck, Germany) for 30 min under slight agitation. Each well was washed with PBS, and the plates were photographed. Finally, crystal violet was eluted in 200 μl of methanol, and absorbance at the wavelength of 595 nm was measured using Spectra Max ABS Plus (Molecular Devices, CA, EUA).

### Statistical Analysis

All experiments were performed at least three times and data were expressed as the mean ± SD. GraphPad Prism 5 (GraphPad Software, CA, USA) was used to perform all statistical analyses. When comparing only two groups, the unpaired Student’s t-test was performed, while for comparison between multiple test groups, One-way analysis of variance (ANOVA) followed by Tukey’s post-test was applied. Spearman’s rank correlation was used for gene expression correlation analyzes. The statistical significance of the overall survival study was determined using the log-rank test. The differences were considered significant whenever P ≤ 0.05.

## Results

### EGFR Expression Positively Correlates With the Expression of Elements of the PAF Pathway in Cervical Cancer Samples

**Figure 2:**
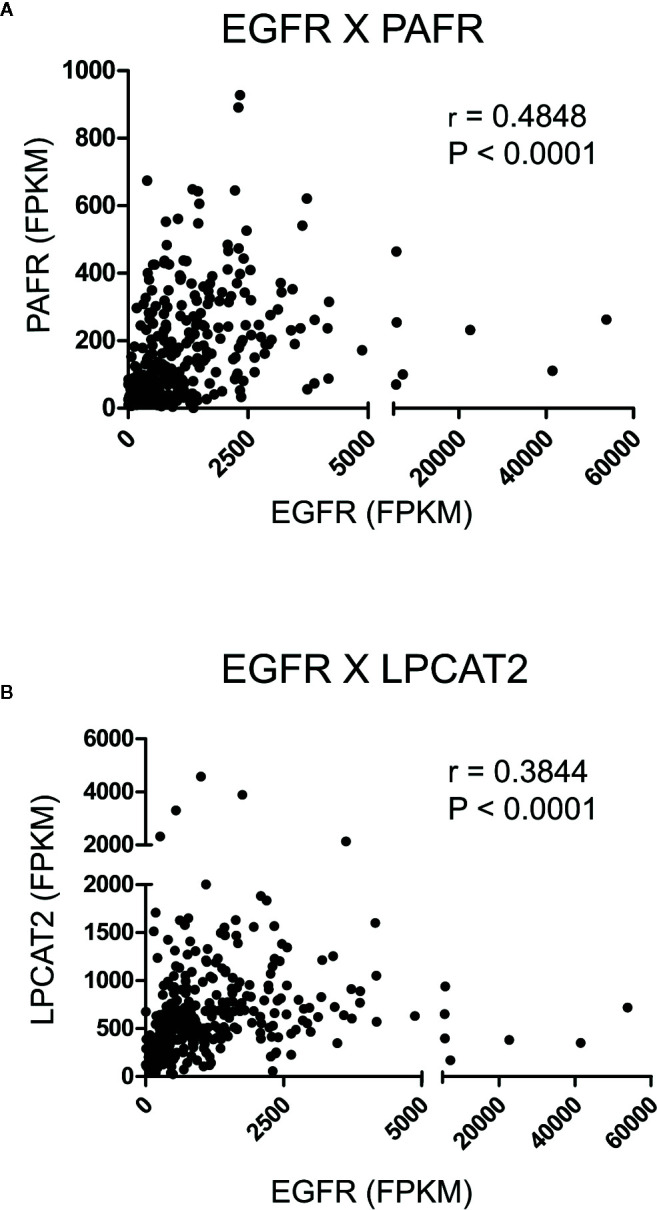
Epidermal growth factor receptor (EGFR) expression correlates with the expression of platelet-activating factor receptor (PAFR) and lysophosphatidylcholine acyltransferase 2 (LPCAT2) in cervical cancer samples. Analyzes of the correlations between EGFR × PAFR **(A)**, and EGFR × LPCAT2 **(B)** in 306 cervical cancer samples. The Cancer Genome Atlas (TCGA) database was used to obtain RNA-seq data, and Spearman’s test was performed to evaluate the coefficient of correlation (r) between the gene expression levels.

To initiate the current study, transcriptome data from cervical cancer patients deposited in TCGA were used to observe possible correlations between the mRNA expression levels of EGFR, cPLA2, PAFR and the components of the LPCAT family. A strong positive correlation was observed between EGFR × PAFR (Spearman’s r = 0.4848, P < 0.0001, **Figure 2A**), and EGFR × LPCAT2 (Spearman’s r = 0.3844, P < 0.0001, **Figure 2B**). No correlation between the expression of EGFR and LPCAT1, LPCAT4 or cPLA2 (**Supplementary Figures 1A, C, D**) was found. Albeit a slight correlation between the expression of EGFR and LPCAT3 was found (Spearman’s r = 0.1160, P = 0.0416, **Supplementary Figure 1B**), PAFR and LPCAT2 were chosen for the subsequent studies due to the strength of the correlation with EGFR, which suggests a possible mechanism of transcriptional regulation.

### LPCAT2 Upregulation Is Associated With Unfavorable Clinical Outcome in Cervical Cancer Patients

**Figure 3:**
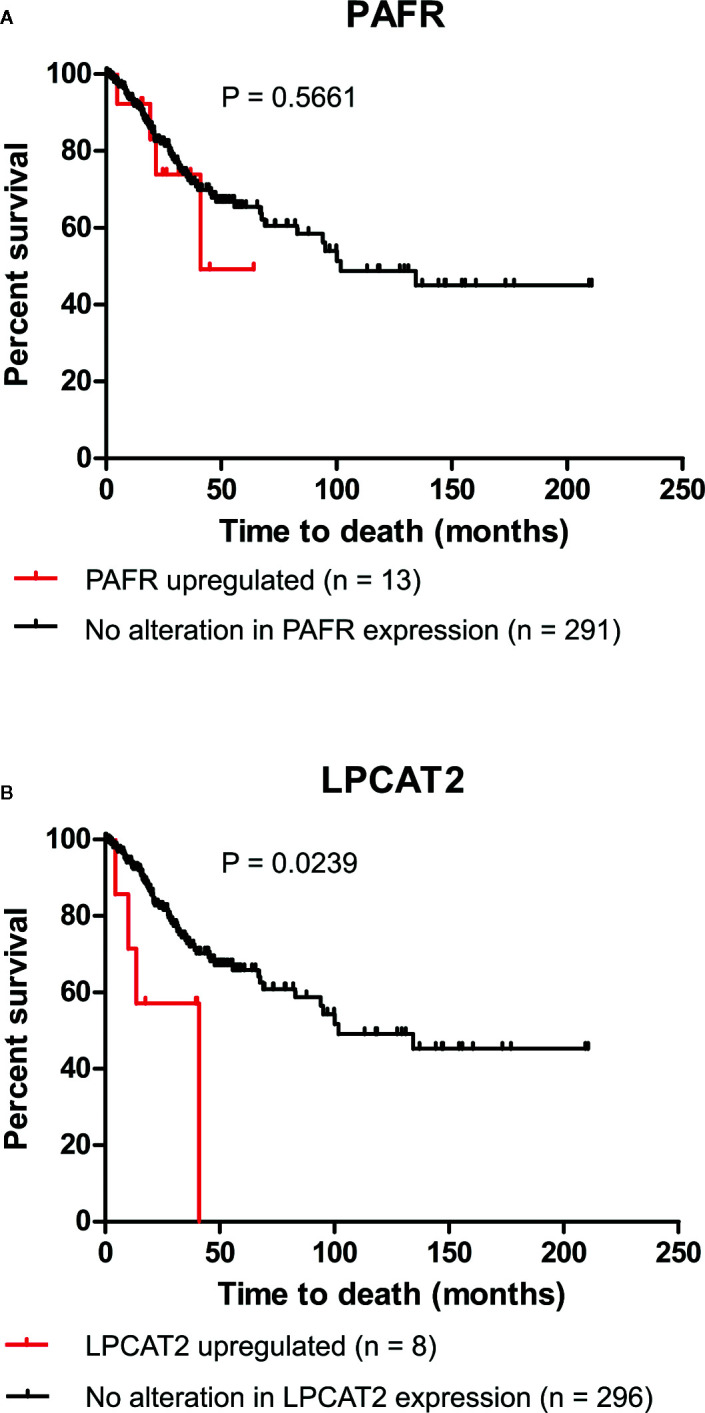
Lysophosphatidylcholine acyltransferase 2 (LPCAT2) upregulation is associated with poor overall survival in cervical cancer patients. Through the cBioPortal platform, were performed overall survival analyzes to evaluate the relation between the upregulations of platelet-activating factor receptor (PAFR) **(A)** and LPCAT2 **(B)**, and prognosis in cervical cancer [The Cancer Genome Atlas (TCGA), Firehose Legacy study; n = 304]. Kaplan-Meier method was implemented to generate survival curves and statistical significance among these curves was determined using the log-rank test.

Recent studies, including ours, have demonstrated that the overexpression of EGFR negatively impacts the overall survival of cervical cancer patients and the response to chemoradiation (6, 11). For this reason, we decided to analyze if the upregulation of elements of the PAF pathway would also negatively affect the overall survival of those patients. Data from 304 patients are available in the cBioPortal platform (TCGA, Firehose Legacy). In the selected criteria, mRNA upregulation of *EGFR*, PAFR (*PTAFR* gene), *LPCAT1*, *LPCAT2*, *LPCAT3*, *LPCAT4*, and cPLA2 (*PLA2G4A* gene) was found in 7.9%, 4.3%, 4.3%, 2.6%, 5.6%, 4.9%, and 3% of the cases, respectively, as shown in **Table 1**. No significant impact on overall survival was observed with increased expression of PAFR (**Figure 3A**), LPCAT1 (**Supplementary Figure 2A**), LPCAT3 (**Supplementary Figure 2B**), and LPCAT4 (**Supplementary Figure 2C**). However, the upregulation of LPCAT2 (P = 0.0239, **Figure 3B**), the same enzyme that positively correlates with the expression of EGFR, showed negative prognostic value for cervical cancer patients, as well as cPLA2 (P = 0.0052, **Supplementary Figure 2D**). The median survival for the group with LPCAT2 overexpressed was 40.9 months vs. 101.7 months for the group without alteration in this gene, thus solidifying the importance of the present study. The prognostic impact of LPCAT2 and cPLA2 must be confirmed in other cohorts of cervical cancer patients since the increased expression of these genes was observed in a small number of cases (8/304 and 9/304, respectively).

**Table 1:** Cases of cervical cancer patients with increased expression of the genes listed (TCGA, Firehose Legacy study, n = 304).

	Cases with mRNA upregulated (%)
EGFR	24/304 (7.9%)
PAFR	13/304 (4.3%)
LPCAT1	13/304 (4.3%)
LPCAT2	8/304 (2.6%)
LPCAT3	17/304 (5.6%)
LPCAT4	15/304 (4.9%)
cPLA2	9/304 (3%)

### EGFR Activation Upregulates the mRNA Expression of Components of the PAF Pathway in Cervical Cancer Cells

For this study, two cervical carcinoma cell lines were used. While the C33A cell line does not exhibit the HPV infection, the CASKI cell line – derived from an epidermoid carcinoma of the cervix metastatic to the small bowel mesentery – shows multiple copies of HPV-16 integrated into its genome. Also, CASKI cells are more resistant to chemoradiation than C33A cells (24). Thus, the CASKI cell line presents a more aggressive behavior than C33A cells, representing a useful and straightforward dichotomic model.

**Figure 4:**
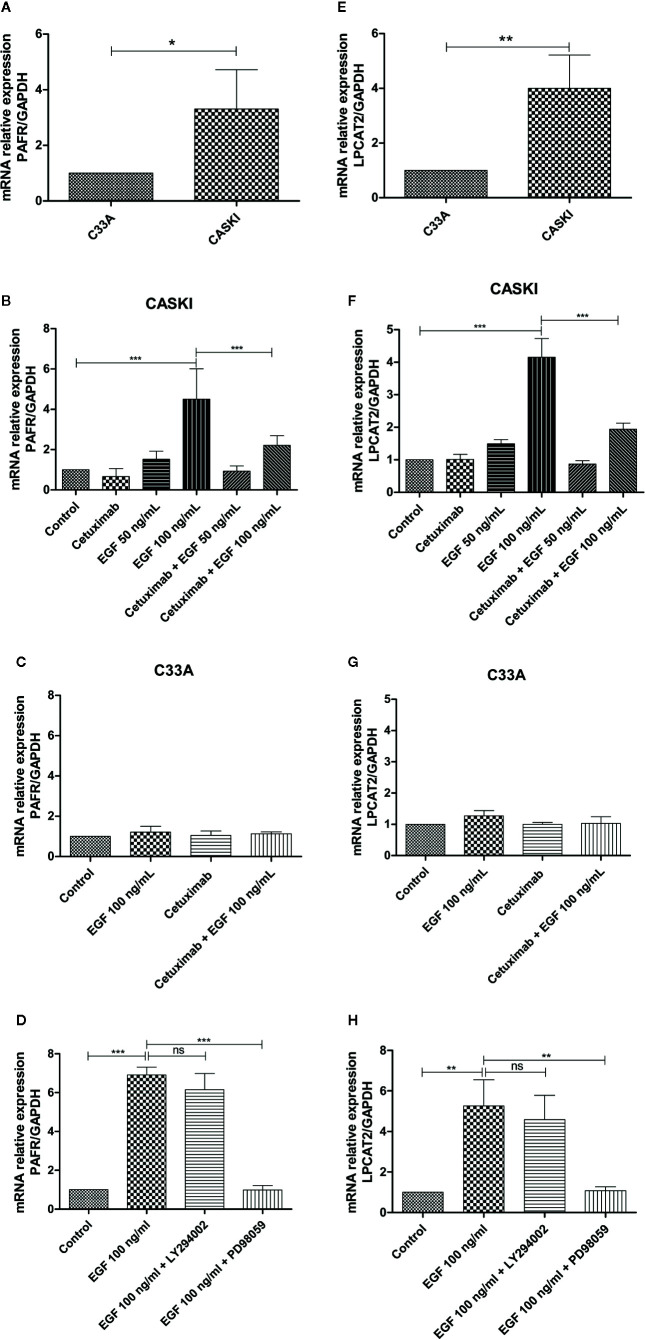
Epidermal growth factor receptor (EGFR) activation upregulates the mRNA expression of lysophosphatidylcholine acyltransferase 2 (LPCAT2) and platelet-activating factor receptor (PAFR) in cervical cancer cells. For the basal expression analysis, qPCR was performed to analyze the relative gene expression of PAFR **(A)** and LPCAT2 **(E)**. To analyze if the activation of EGFR modulates components of the PAF pathway, cells were starved for 16 h, and then treated with cetuximab (100 µg/ml). One hour and a half later, cells were stimulated with EGF (50 or 100 ng/ml) for 1.5 h. After treatment, mRNA was extracted and converted into cDNA. Gene expression assays for PAFR in CASKI cells **(B)** and C33A cells **(C)**; and for LPCAT2 in CASKI cells **(F)** and C33A **(G)** were performed. Alternatively, CASKI cells were starved and pretreated with PD98059 (50 µM) or LY294002 (25 µM) for 1.5 h. Then, cells were stimulated with EGF (100 ng/ml) for 1.5 h. Gene expression assays for PAFR **(D)** and LPCAT2 **(H)** were performed. The comparative CT method (ΔΔCT) was utilized to calculate relative mRNA expression and GAPDH was used as the reference gene. Values represent mean + SD of at least three independent experiments. For multiple comparisons, one-way ANOVA and Tukey’s post-test were used; while for comparisons of only two conditions, Student’s t-test was performed. ns, not significant, *P < 0.05, **P < 0.01, ***P < 0.001.

A previous study from our group showed that CASKI cells express about 80 times more EGFR than the C33A cell line. This difference is also reflected at the protein level since C33A cells showed undetectable levels of EGFR by western blotting (11). To observe if this pattern is maintained for the expression of PAFR and LPCAT2, quantitative PCR was performed. As seen for EGFR, CASKI cells present higher basal mRNA expression levels of PAFR (**Figure 4A**) and LPCAT2 (**Figure 4E**) than C33A cells.

Stimulation of EGFR has been associated with the activation of the PAFR pathway (19). In this context, we examined whether the treatment with EGF would stimulate the expression of PAFR and LPCAT2. CASKI cells were incubated with EGF (50 ng/ml or 100 ng/ml) and mRNA levels of PAFR (**Figure 4B**) and LPCAT2 (**Figure 4F**) were again measured by qPCR. Stimulation with the highest concentration of EGF was able to increase the expression of both elements of the PAF pathway. Moreover, pre-incubation with cetuximab, a potent monoclonal antibody against EGFR, was able to block the effects of EGF over the cells. Not surprisingly, stimulation of C33A with the highest concentration of EGF was not able to elevate mRNA levels of either PAFR (**Figure 4C**) or LPCAT2 (**Figure 4G**). These results indicate that the activation of EGFR can positively modulate the expression of the PAF receptor and the LPCAT2 enzyme, possibly elevating PAF production in aggressive cervical cancer cells. Then, we decided to identify the contribution of EGFR-activated classically signaling pathways in the upregulation of PAFR and LPCAT2. The Ras-Raf-MEK-ERK and PI3K-Akt-mTOR signaling pathways were inhibited with PD98059 (MEK inhibitor) and LY294002 (PI3K inhibitor), respectively. Interestingly, PD98059 completely reversed EGF-mediated PAFR (**Figure 4D**) and LPCAT2 (**Figure 4H**) induction in CASKI cells, unlike LY294002. These results suggest that the ERK/MAPK pathway is responsible for modulating the expression of these genes after EGFR activation.

### PAF Induces the Transactivation of the EGFR and the Downstream Signaling Pathways

**Figure 5:**
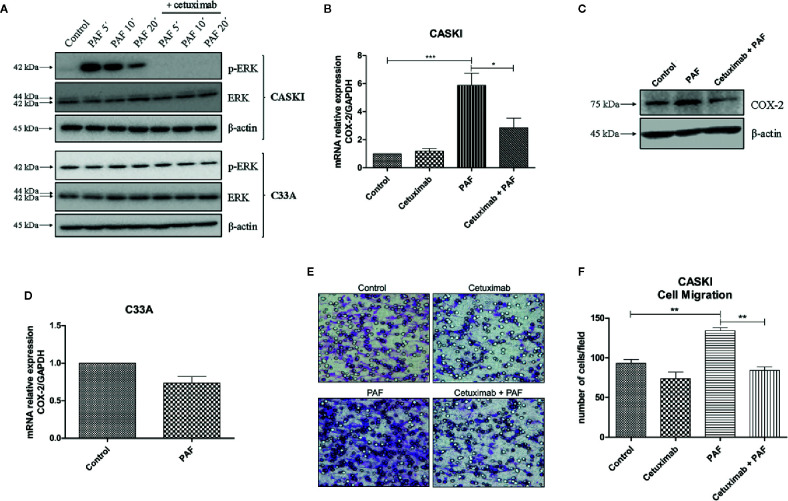
Platelet-activating factor (PAF) transactivates epidermal growth factor receptor (EGFR), modulating MAPK activation, COX-2 upregulation, and cell migration. **(A)** CASKI and C33A cells were starved for 16 h and treated with cetuximab (100 µg/ml). One and a half-hour later, cells were stimulated with PAF (100 nM) for 5–20 min. Afterward, cells were lysed and levels of p-ERK, total ERK and beta-actin (loading control) were determined by Western blotting. Representative image of two experiments. **(B)** After starving, CASKI cells were treated with cetuximab (100 µg/ml) for 1.5 h, and subsequently treated with PAF (100 nM) also for 1.5 h. Then, mRNA was extracted and converted into cDNA. Gene expression assays for COX-2 and GAPDH (reference gene) were performed by qPCR. Values represent mean + SD of five independent experiments. **(C)** CASKI cell line was starved and treated with cetuximab (100 µg/ml) for 1.5 h, and subsequently stimulated with PAF (100 nM) for 3 h, when cells were lysed and the levels of COX-2 and β-actin (loading control) were determined by Western blotting. Representative image of two experiments. **(D)** C33A cells were starved and stimulated with PAF (100 nM) for 1.5h. Gene expression assays for COX-2 and GAPDH were also performed. Values represent mean + SD of five independent experiments. **(E, F)** For the cell migration assay, after starving, CASKI cells were treated with cetuximab (100 µg/ml) for 1.5 h followed by 16 h stimulation with PAF (100 nM). Then, cells were added to a Boyden chamber, using 8 μm pore polycarbonate membranes, and left to migrate toward RPMI containing 5% SFB for 20 h. **(E)** Representative images of the migration assay are shown on the panel (100× magnification). **(F)** Values represent mean of the number of cells migrated counted per field + SD of three independent experiments. For multiple comparisons, one-way ANOVA and Tukey’s post-test were used; while for comparisons of only two conditions, Student´s t-test was performed. *P < 0.05, **P < 0.01, ***P < 0.001.

In ovarian cancer models, it has been shown that the stimulation of PAFR transactivates EGFR (20, 21). In this context, we examined if the same was true for the cervical cancer model. CASKI cells were incubated with PAF, for different periods of time, and the ERK1/2 phosphorylation was further assessed since the MAPK pathway is a known downstream route of the EGFR. The activation of PAFR caused a significant increase in ERK phosphorylation that was completely blocked by pre-incubation with cetuximab (**Figure 5A**). As already shown by our group, C33A cells have higher basal levels of p-ERK1/2 than the CASKI cell line (24), probably due to a loss-of-function mutation in the *PTEN* gene (25). Interestingly, PAF failed to induce ERK activation in C33A (**Figure 5A**).

This same pattern of biological response was observed in the expression of cyclooxygenase-2 (COX-2), a downstream gene of the EGFR signaling (26, **Supplementary Figure 3**). COX-2 is an inflammatory enzyme overexpressed in cervical tumors (26), with negative prognostic value in this type of cancer (11, 27). PAFR activation increased COX-2 expression in CASKI cells, and pre-incubation with cetuximab blocked this phenomenon, both at the mRNA (**Figure 5B**) and protein levels (**Figure 5C**). In C33A cells, COX-2 expression is not modulated by PAF stimuli (**Figure 5D**), as well MAPK activation, possibly due to its lower levels of PAFR (mRNA and protein) as compared to CASKI cells (**Figure 4A** and **Supplementary Figure 4A**), in addition to not expressing EGFR. These results indicate that the effects observed upon activation of the PAF-PAFR axis are mostly dependent on EGFR activity.

Interestingly, in addition to blocking PAF-mediated EGFR transactivation, treatment with cetuximab for 48 h downregulated PAFR expression by 35% (**Supplementary Figure 4B**). This represents another mechanism for attenuating PAF signaling, corroborating our findings that EGFR positively regulates PAFR expression (**Figure 4B**).

Beyond the analysis of the impact of the EGFR transactivation by PAF in signaling cascades and gene expression modulation, we analyzed its effect on CASKI cells’ ability to migrate. PAF promoted an increase of the motility of this cell line, a phenomenon that was completely reversed by EGFR inhibition with cetuximab (**Figures 5E, F**). Since treatment with cetuximab alone showed no difference from the control and that the pre-incubation with cetuximab reversed the PAF effects, our results again suggest that EGFR is the downstream effector of PAFR in CASKI cells.

### Combined Inhibition of PAFR and EGFR Impairs the Clonogenic Capacity of Aggressive Cervical Cancer Cells

Since a positive feedback loop appears to occur in CASKI cells, where EGFR induces the expression of components of the LPCAT-PAF-PAFR axis and PAF can transactivate EGFR, we assessed the biological impact of the combined inhibition of EGFR and PAFR in this cell line. Cetuximab was used to inhibit EGFR, while WEB 2086 was used to inhibit PAFR. WEB 2086 is a very potent, safe, tolerable, and selective PAF antagonist (28).

**Figure 6:**
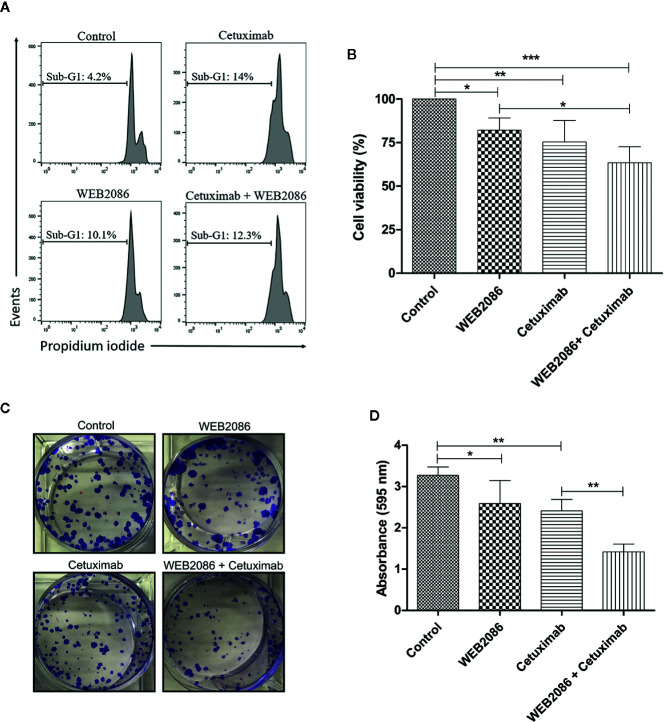
Effects of the combined platelet-activating factor receptor (PAFR) and epidermal growth factor receptor (EGFR) inhibition in CASKI cells. **(A)** CASKI cells were treated with cetuximab (100 µg/ml) and/or WEB 2086 (50 µM) for 24 h. Cell death was evaluated by propidium iodide staining, followed by flow cytometry. Cells with hypodiploid DNA content (sub-G1 peak) were considered apoptotic cells. Representative histograms of three independent experiments. **(B)** For the MTT assay, CASKI cells were seeded at 2,500 cells/well in 96-well cell culture plates. Then, cells were treated with cetuximab (100 μg/ml) and/or WEB 2086 (100 μM) for 72 h in medium supplemented with 2% fetal bovine serum (FBS). After treatment, cells were incubated with a solution of MTT. The formazan crystals generated were solubilized in DMSO and the optical density was measured at a wavelength of 538 nm. Cell viability was expressed as a percentage of the control. Values represent mean + SD of four independent experiments. **(C, D)** For the clonogenic assay, CASKI cells were seeded at low density and treated for 13 days with cetuximab (100 µg/ml) and/or WEB 2086 (50 µM). Then, colonies were stained with crystal violet and photographed. **(C)** Representative image of the colonies of the CASKI cell line after targeted therapies against EGFR and PAFR. **(D)** Crystal violet was eluted in methanol and absorbance at 595 nm was measured. Values represent mean + SD of five independent experiments. One-way ANOVA and Tukey’s post-test were used. *P < 0.05, **P < 0.01, ***P < 0.001.

CASKI cell line was treated with cetuximab and/or WEB 2086 for 24 h, when cell death was evaluated, using propidium iodide staining. DNA fragmentation was measured since it is a key feature of cell death (29). Cells treated with cetuximab or WEB2086 exhibited 10-14% of its events within sub-G1 DNA content, as shown by flow cytometry, indicating cells undergoing DNA fragmentation. However, combined treatment did not present an additive/synergistic effect in cell death, showing 12% of the events with hypodiploid DNA content (**Figure 6A**).

In addition to cell death, we evaluated the effect of combining cetuximab with WEB2086 on the viability of CASKI cells after 72 h of treatment. Isolated treatment with WEB2086 or cetuximab decreased cell viability by approximately 18% and 25%, respectively. Interestingly, the combination of WEB2086 + cetuximab compromised CASKI cells’ viability by about 37% (**Figure 6B**).

Ultimately, the prolonged treatment of CASKI cells (13 days), measured by colony formation assay, revealed an interesting pharmacologic strategy. Combined treatment significantly reduced the size and number of colonies in relation to control and to isolated treatments with cetuximab or WEB2086 (**Figure 6C**), causing reduced staining of the colonies with crystal violet, as evidenced in the absorbance of the eluted dye (**Figure 6D**). These findings indicate that the combined targeting treatment using anti-PAFR and anti-EGFR may have significant therapeutic potential for cervical cancer cells.

## Discussion

In the current study, strong positive correlations between EGFR × PAFR, and EGFR × LPCAT2 in cervical cancer samples were shown, which led to the hypothesis that EGFR can regulate the expression of these genes involved in the biosynthesis and signaling of PAF. Indeed, EGFR activation upregulated the expression of PAFR and LPCAT2 in cervical cancer cells through a MAPK-dependent mechanism.

The PAF-PAFR axis is largely associated with cancer progression (30), neoangiogenesis (31), modulation of the tumor microenvironment with downregulation of the anti-cancer immune response (32), and evasion of the chemoradiation-induced cell death (16, 33). To our knowledge, there is only one study that investigated the role of PAFR in cervical cancer, where increased expression of this receptor was observed in tumor samples as compared to normal cervical tissues (33). In this same study, authors observed higher PAFR expression in cervical tumors from patients who underwent irradiation compared with biopsies taken before radiotherapy. Moreover, radiotherapy increased PAFR expression *in vitro* and induced the production of PAF-like molecules in cervical cancer cells. Finally, the PAFR blockade sensitized these tumor cell lines to γ-radiation (33).

To date, few studies have investigated the function of LPCAT2 in tumor biology. The LPCAT2 expression levels were positively correlated with aggressive behavior in prostate cancer (34) and were significantly upregulated in cervical, breast, and colon cancer tissues, suggesting a role in the progression of these tumors (35). Recently, Cotte and colleagues showed that LPCAT2 drives cell-death resistance to chemotherapy through a lipid droplet accumulation-dependent mechanism, impairing caspase activation and endoplasmic reticulum stress response (36). Our results revealed that the LPCAT2 upregulation, but not PAFR, has negative prognostic value in cervical cancer patients. In accordance with our study, Deng and colleagues showed the PAF exacerbates peritonitis partly through inflammasome activation, but PAFR is dispensable for PAF-induced inflammasome activation in vivo or in vitro (37). These data, along with ours, suggest that the presence of PAF (generated by LPCAT2, for example) is more important for the aggressive behavior of some tumor types than the levels of PAFR expressed in the cancer cells.

Abnormal phosphatidylcholine (PC) metabolism is reported in cervical cancer. A recent study identified that PC and lyso-PC (also known as lyso-PAF) are down- and upregulated in plasma of cervical cancer patients, respectively, compared to patients with the benign uterine disease (38). These results suggest increased activity of the PLA2 enzyme in cervical cancer since the hydrolysis of PC by PLA2 generates both lyso-PC and free fatty acids. It remains to be determined whether lyso-PC is only a metabolic intermediate for LPCATs or has a role in cancer cell signaling. Some lyso-PC species might be an important function in Chagas disease progression (39). Interestingly, we showed that increased expression of cPLA2 is associated with poor overall survival in cervical cancer patients. However, EGFR expression did not correlate with cPLA2 expression levels in cervical cancer samples. An interesting hypothesis is that EGFR signaling pathways regulate cPLA2 at post-translational level, rather than gene expression regulation since this PLA2 isoform is activated by phosphorylation and Ca^2+^ (18).

Several studies have shown that GPCRs can transactivate EGFR in different models (9–11). However, PAFR-mediated EGFR transactivation has only been demonstrated in ovarian cancer cells so far (20, 21). In this ovarian cancer model, EGFR transactivation involves the PAFR activation, the activation of phospholipase C-β, inositol trisphosphate-induced Ca^2+^ mobilization, activation of the non-receptor tyrosine kinase Src, cleavage and secretion of heparin-binding EGF-like growth factor (HB-EGF) in a matrix metalloproteinase-dependent mechanism (21).

Alternatively, EGFR can be transactivated by GPCRs in the absence of EGF-like ligands, suggesting that the EGFR transactivation by GPCRs can also occur through intracellular signaling routes (40). In our study, we observed that the PAF-induced ERK1/2 phosphorylation was completely inhibited by the anti-EGFR monoclonal antibody, cetuximab, in CASKI cells. Cetuximab blocks EGFR activation because it binds with high affinity to the receptor’s extracellular domain, preventing interaction with physiological ligands (41). Thus, our results suggest that EGFR transactivation by PAF occurs through the shedding of EGF-like ligands in the extracellular milieu.

In CASKI cells, EGFR transactivation by PAF induced the expression of the enzyme COX-2. High expression of COX-2 and increased production of its major metabolite, PGE2, have been found in the cervical carcinoma in relation to normal cervix (42). Kim and colleagues (27) showed that the coexpression of EGFR and COX-2 may be used as a potent risk factor to predict the poor survival of patients with squamous cell carcinoma of the uterine cervix. PGE2 is the most abundant prostaglandin in humans and is known as a critical mediator in inflammation. The functions of PGE2 are mainly facilitated by four specific G-protein-coupled receptors (EP1-EP4) with various signaling pathways (43). Sales and colleagues reported that, in addition to the expression of COX-2 and production of PGE2, cervical tumors express EP2 and EP4 receptors, suggesting an autocrine/paracrine regulation of the neoplastic cell function (42). Jung-Min et al. showed that HPV16 oncoproteins induce EP4 receptor expression in cervical cancer cells (44). In this same study, the authors showed an increased expression of EP4 in 52 cervical cancer tissues compared with four healthy controls by immunohistochemistry (44). EP4 plays a role in cervical cancer progression since GW627368X (a highly selective EP4 antagonist) inhibits the proliferation and angiogenesis of cervical cancer cell lines and suppresses tumor growth in xenograft mice model (45). In addition, EP3 upregulation was associated with poor overall survival of 250 cervical cancer patients. The EP3 receptor was also significantly correlated with lymph node invasion and tumor staging (46). It remains to be determined whether COX-2-derived prostanoids have pro-tumor effects in our model and whether PAF can induce the expression of PGE2 receptors, especially EP2, EP3 and EP4.

**Figure 7:**
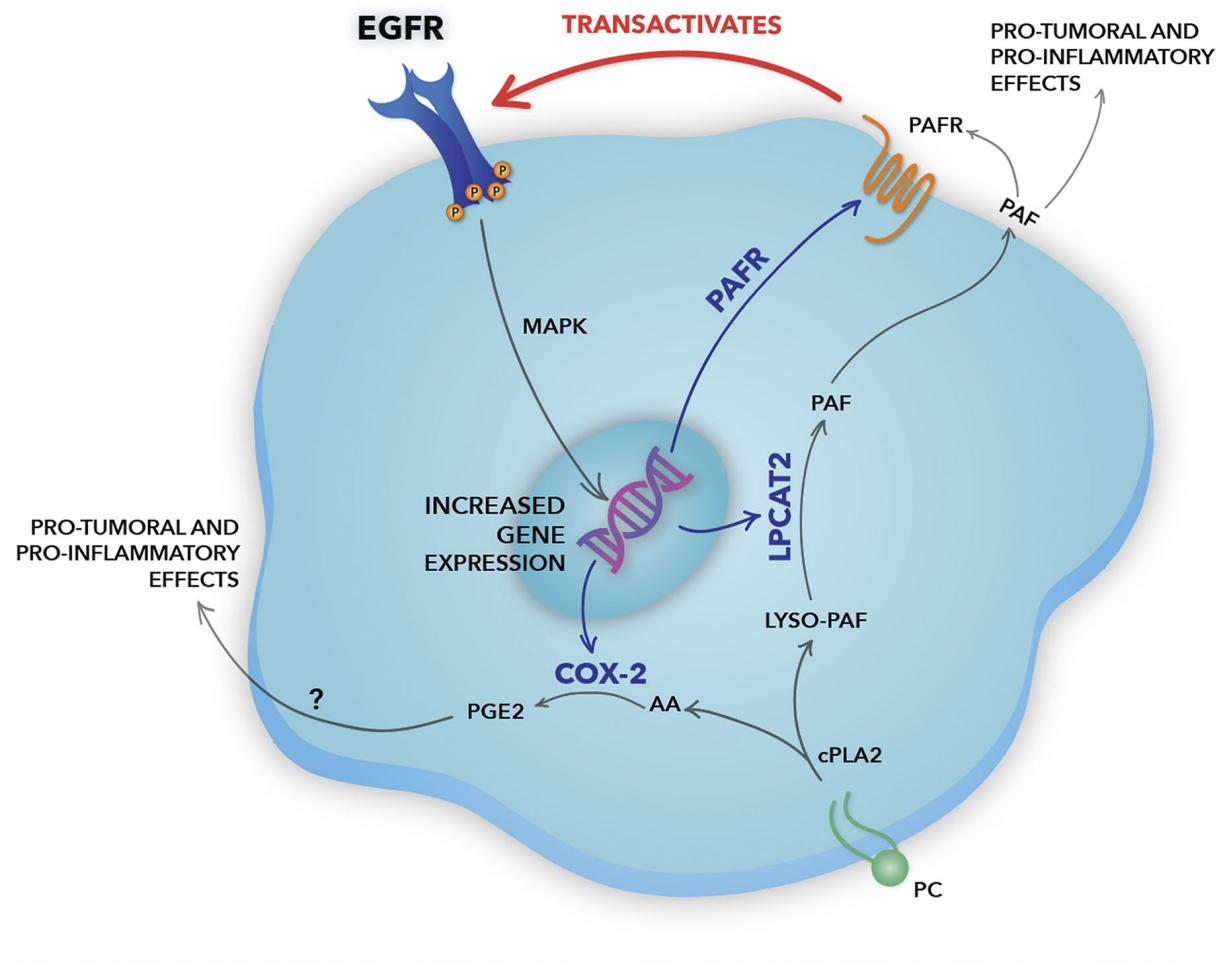
Schematic representation of the positive crosstalk between epidermal growth factor receptor (EGFR) and the platelet-activating factor (PAF) pathway in cervical cancer cells. In our hypothesized model, EGFR activation induces gene expression of the lysophosphatidylcholine acyltransferase 2 (LPCAT2) enzyme, a member of the PAF biosynthesis pathway. EGFR can also induce the expression of PAF receptor (PAFR), which, then, can be activated by the PAF produced by LPCAT2 in EGFR-expressing cancer cells, in an autocrine fashion, or surrounding cells in a paracrine mechanism. PAFR can induce EGFR transactivation through the cleavage and release of EGF-like ligands from the plasma membrane, promoting MAPK activation and cell migration. The bidirectional interaction between the EGFR and the LPCAT-PAF-PAFR axis reveals the presence of a positive feedback loop. Still, the activation of both EGFR and PAFR leads to the expression of the inflammatory enzyme COX-2. COX-2 produces prostanoids, such as prostaglandin E2 (PGE2), using arachidonic acid released after the enzymatic action of cPLA2, the same enzyme that initiates the PAF biosynthesis pathway. Studies have shown that PGE2, like PAF, has an important pro-inflammatory and pro-tumoral effect, being able to transactivate EGFR. Genes upregulated by EGFR are indicated by blue arrows. Therefore, EGFR rises as a central pillar of the signaling pathways mediated by GPCRs, inducing a more aggressive phenotype in cervical cancer cells.

In the hypothesized model (**Figure 7**), the EGFR activation induces the expression of the LPCAT2 enzyme, a member of the remodeling pathway of PAF biosynthesis. Besides, EGFR can induce the expression of the PAF receptor, which can be activated by the PAF produced by LPCAT2 in EGFR-expressing cancer cells, in an autocrine mechanism, or surrounding cells in the microenvironment, in a paracrine fashion. PAFR, then, can transactivate EGFR through the cleavage and release of EGF-like ligands from the plasma membrane. EGFR activation promotes LPCAT2 upregulation, and consequently PAF production and PAFR activation, suggesting a positive feedback loop. Still, the activation of both EGFR and PAFR leads to the expression of the inflammatory enzyme COX-2. COX-2 produces prostanoids such as prostaglandin E2 (PGE2), using arachidonic acid released after the enzymatic action of cytosolic PLA2 (43), the same enzyme that initiates the PAF production pathway. Therefore, in situations in which PGE2 is produced, depending on the levels of LPCATs expression, PAF is also produced. PGE2, as well as PAF, is a lipid mediator with potent pro-tumoral and pro-inflammatory activities (43). Studies have shown that PGE2 can also transactivate EGFR through EP receptors (47). This positive crosstalk between the PAF-PAFR axis and EGFR demonstrates an important linkage between inflammatory and growth factor signaling in cervical cancer cells.

We also showed that the combined inhibition of EGFR and PAFR has great therapeutic potential in cervical cancer cells that express these receptors. However, a dissonance was observed between the results of the short-term cell death assay and the long-term colony formation assay, which lasts for 13 days. It is important to highlight that the anti-cancer therapeutic agents not only induce apoptotic cell death but also trigger growth-arresting events, such as other mechanisms of cell death, quiescence, accelerated senescence and mitotic catastrophe, responses that are manifested several days after the introduction of the treatment. Thus, the colony formation assay provides an integrated readout of all these early and late responses, being considered the gold standard for cytotoxicity evaluation (48).

Further investigation into the potential use of EGFR inhibitors, PAF antagonists and LPCAT inhibitors in the treatment of cervical cancer is needed through preclinical and clinical studies. Remarkably, we must emphasize the importance of an adequate selection of patients for a future clinical trial involving these target drugs, since only 26% of the cervical cancer patients included in TCGA (Firehose Legacy study) showed upregulation of, at least, one of the genes of these signaling pathways (*EGFR*, *PTAFR*, *LPCAT1*, *LPCAT2*, *LPCAT3*, *LPCAT4*, and *PLA2G4A*).

In the current study, we showed a significant positive correlation between EGFR and PAFR, and between EGFR and LPCAT2 in 306 cervical cancer samples deposited in TCGA database. In these patients, LPCAT2 upregulation was associated with poor prognosis. Moreover, we also reported an interplay between the EGFR signaling pathway and components of the LPCAT-PAF-PAFR axis in cervical cancer cells. Finally, combined EGFR and PAFR inhibition compromised cell viability and clonogenic capacity of aggressive cervical cancer cells. Taken together, our data suggest that EGFR, LPCAT2, and PAFR emerge as novel targets for cervical cancer therapy.

## Data Availability Statement

The datasets presented in this study can be found in online repositories. The names of the repository/repositories and accession number(s) can be found below: The ‘Cervical Squamous Cell Carcinoma and Endocervical Adenocarcinoma (TCGA, Firehose Legacy)’ study data from cBioPortal [https://www.cbioportal.org/study/summary?id=cesc_tcga].

## Author Contributions

JS performed and analyzed the experiments, wrote the manuscript. KM-C and IG performed experiments. AM and AL analyzed the data and participated in manuscript preparation. RM provided the hypothesis question, wrote the manuscript, and analyzed the data. VA performed and analyzed the experiments, wrote the manuscript, designed the study, and provided the hypothesis question. All authors contributed to the article and approved the submitted version.

## Funding

This work was supported by the Brazilian National Council for Scientific and Technological Development (CNPq) [309946/2018-2]; the State of Rio de Janeiro Research Foundation (FAPERJ) [E-26/202.871/2018]; and the Coordination for the Improvement of Higher Education Personnel (CAPES) [23038.008921/2019-15].

## Conflict of Interest

The authors declare that the research was conducted in the absence of any commercial or financial relationships that could be construed as a potential conflict of interest.

## Footnotes

1
https://cancergenome.nih.gov/

## Supplementary Material

Supplementary Figure 1 Correlation analyzes between the EGFR expression and the expression of cPLA2 and LPCAT enzymes in cervical cancer samples. Analyzes of the correlations between the expression levels of EGFR × LPCAT1 **(A)**, EGFR × LPCAT3 **(B)**, EGFR × LPCAT 4 **(C)**, and EGFR × cPLA2 **(D)** in 306 cervical cancer samples. TCGA database was used to obtain RNA-seq data, and Spearman’s test was performed to evaluate the strength of the correlations.

Supplementary Figure 2 Relation between the increased expression of cPLA2 and LPCAT enzymes with overall survival in cervical cancer patients. Through the cBioPortal platform, overall survival analyzes were performed to evaluate the association between the upregulations of LPCAT1 **(A)**, LPCAT3 **(B)**, LPCAT4 **(C)**, and cPLA2 **(D)** with prognosis in cervical cancer (TCGA, Firehose Legacy study; n = 304). Kaplan-Meier method was used to generate survival curves, and the statistical significance was determined using the log-rank test.

Supplementary Figure 3 EGFR upregulates COX-2 expression in CASKI cells. After starving, CASKI cells were treated with cetuximab (100 µg/ml) for 1.5 h and subsequently treated with EGF (100 ng/ml) for 1.5 h. Then, mRNA was extracted and converted into cDNA. Gene expression assays for COX-2 and GAPDH (reference gene) were performed by qPCR. Values represent mean + SD of three independent experiments.

Supplementary Figure 4 PAFR protein levels are differentially expressed in cervical cancer cell lines and regulated by EGFR. **(A)** CASKI and C33A cell lines were lysed and the levels of PAFR and β-actin (loading control) were determined by Western blotting. Representative image of three experiments. **(B)** CASKI was treated with cetuximab (100 µg/ml) for 24 h or 48 h in serum-free medium, when cells were lysed and the levels of PAFR and β-actin (loading control) were determined by Western blotting.

## Abbreviations

COX-2, cyclooxygenase 2; cPLA2, cytosolic phospholipase A2; EGF, epidermal growth factor; EGFR, epidermal growth factor receptor; ERK, extracellular signal-regulated kinase; GAPDH, glyceraldehyde 3-phosphate dehydrogenase; GPCR, G protein-coupled receptor; HPV, human papillomavirus; LPCAT, lysophosphatidylcholine acyltransferase; MAPK, mitogen-activated protein kinase; PAF, platelet-activating factor; PAFR, platelet-activating factor receptor; PC, phosphatidylcholine; PGE2, prostaglandin E2; TCGA, The Cancer Genome Atlas.
