# Supplementary figures and images for: Interplay Between EGFR and the Platelet-Activating Factor/PAF Receptor Signaling Axis Mediates Aggressive Behavior of Cervical Cancer
Source: Front Oncol. 2020 Dec 17;10:557280. doi: 10.3389/fonc.2020.557280 (PMC7773908; doi:10.3389/fonc.2020.557280)

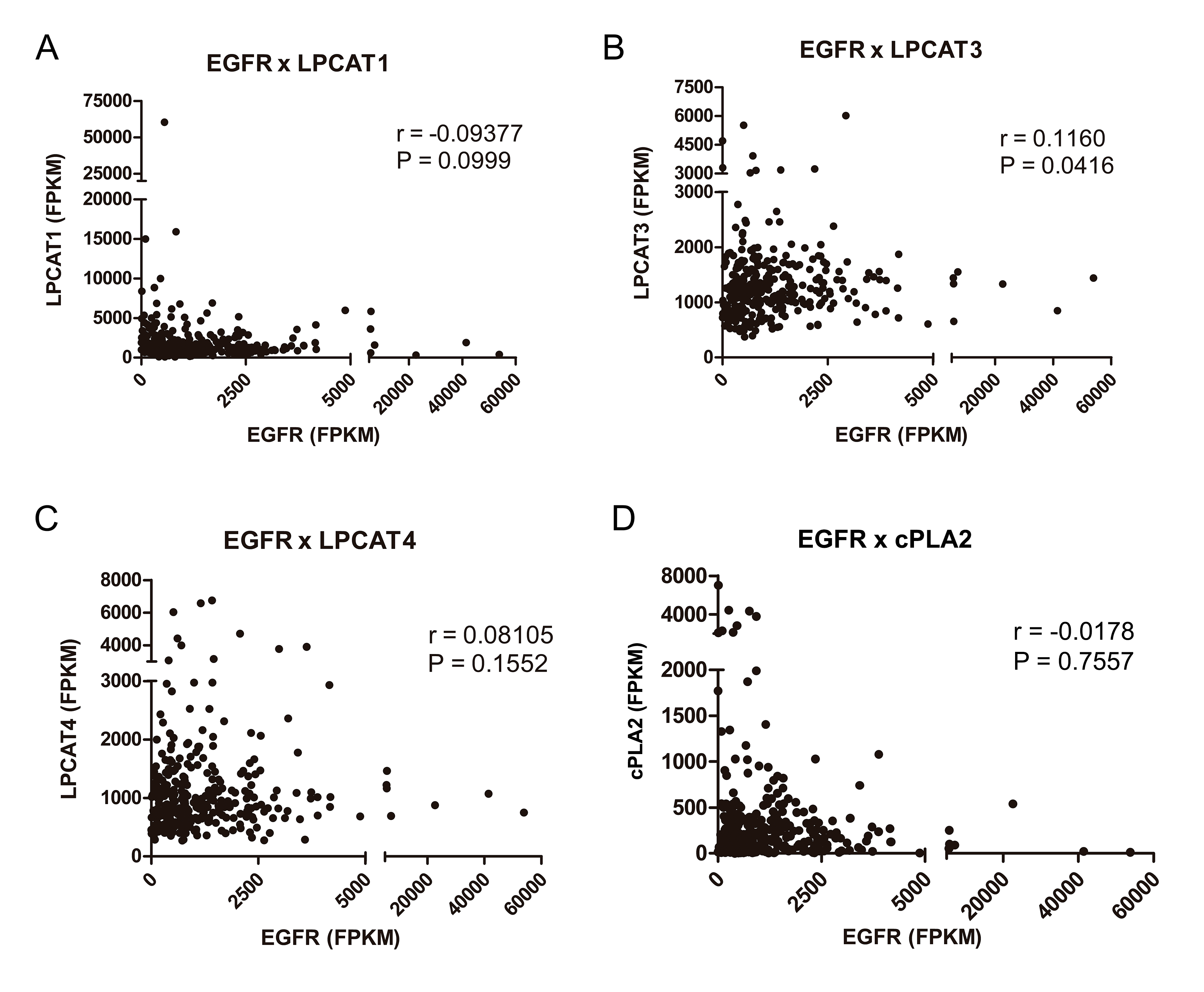

Supplement: Supplementary Figure 1 — Correlation analyzes between the EGFR expression and the expression of cPLA2 and LPCAT enzymes in cervical cancer samples. Analyzes of the correlations between the expression levels of EGFR × LPCAT1 (A), EGFR × LPCAT3 (B), EGFR × LPCAT 4 (C), and EGFR × cPLA2 (D) in 306 cervical cancer samples. TCGA database was used to obtain RNA-seq data, and Spearman’s test was performed to evaluate the strength of the correlations. [file Image_1.tif]

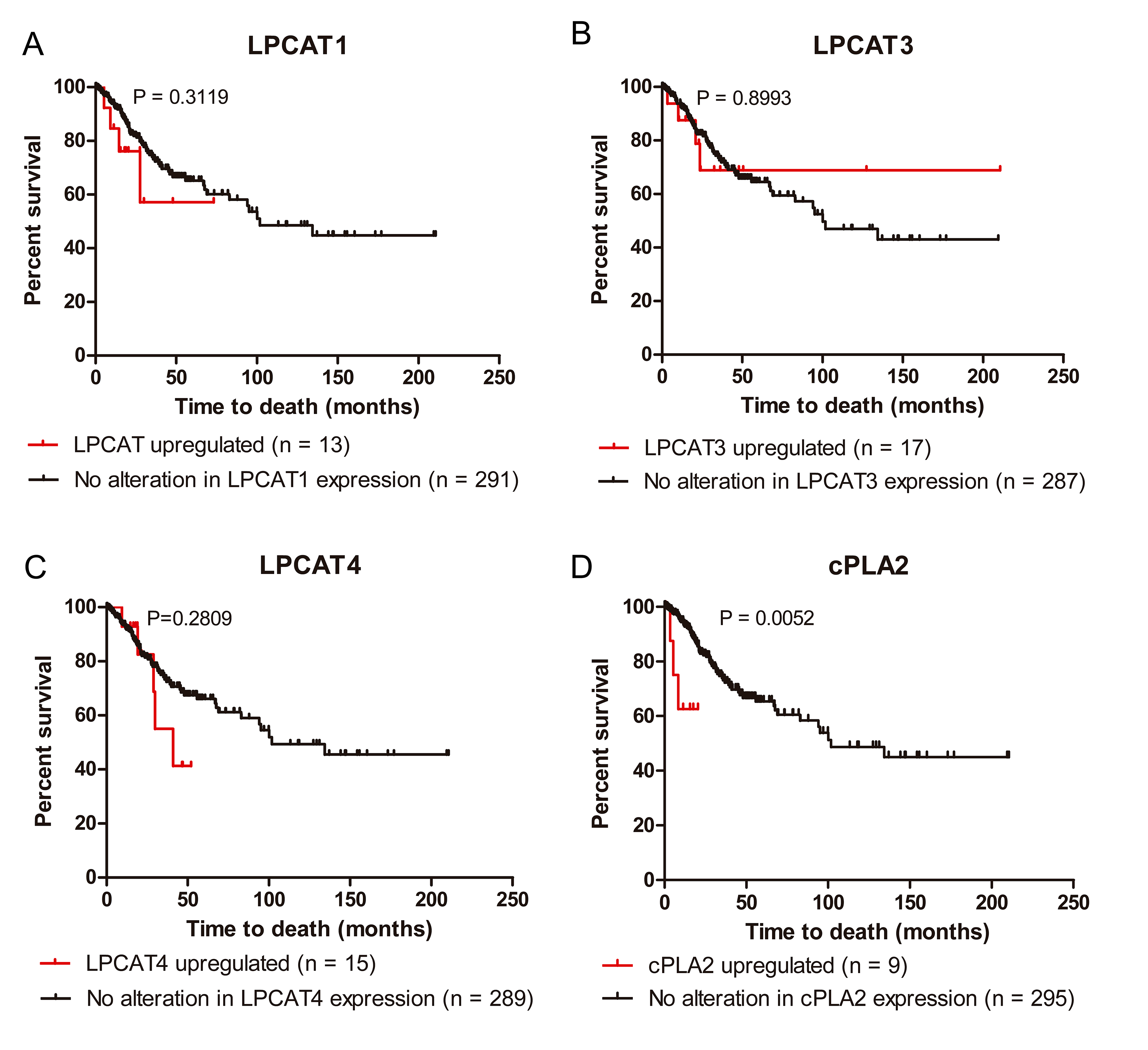

Supplement: Supplementary Figure 2 — Relation between the increased expression of cPLA2 and LPCAT enzymes with overall survival in cervical cancer patients. Through the cBioPortal platform, overall survival analyzes were performed to evaluate the association between the upregulations of LPCAT1 (A), LPCAT3 (B), LPCAT4 (C), and cPLA2 (D) with prognosis in cervical cancer (TCGA, Firehose Legacy study; n = 304). Kaplan-Meier method was used to generate survival curves, and the statistical significance was determined using the log-rank test. [file Image_2.tif]

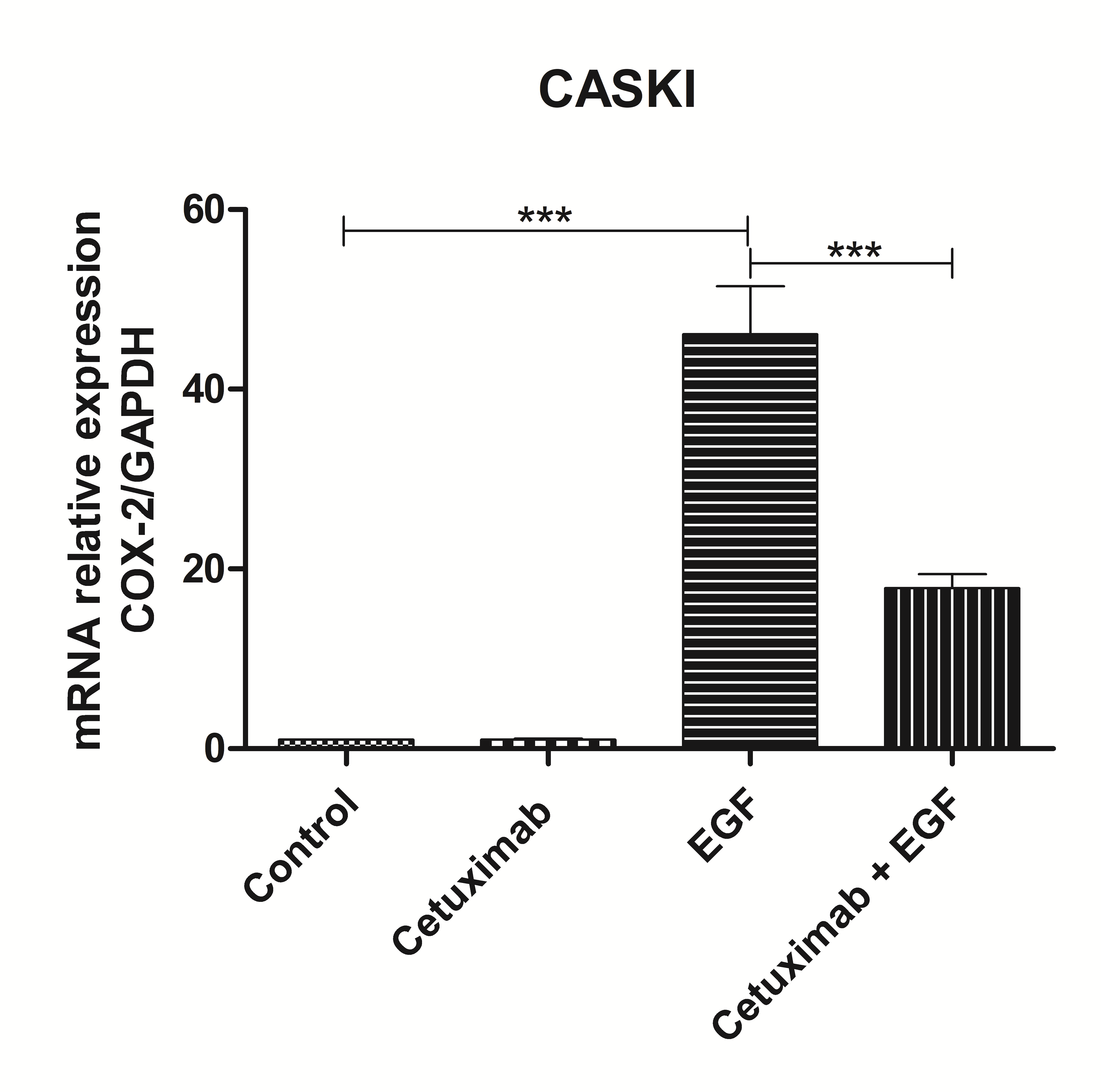

Supplement: Supplementary Figure 3 — EGFR upregulates COX-2 expression in CASKI cells. After starving, CASKI cells were treated with cetuximab (100 µg/ml) for 1.5 h and subsequently treated with EGF (100 ng/ml) for 1.5 h. Then, mRNA was extracted and converted into cDNA. Gene expression assays for COX-2 and GAPDH (reference gene) were performed by qPCR. Values represent mean + SD of three independent experiments. [file Image_3.tif]

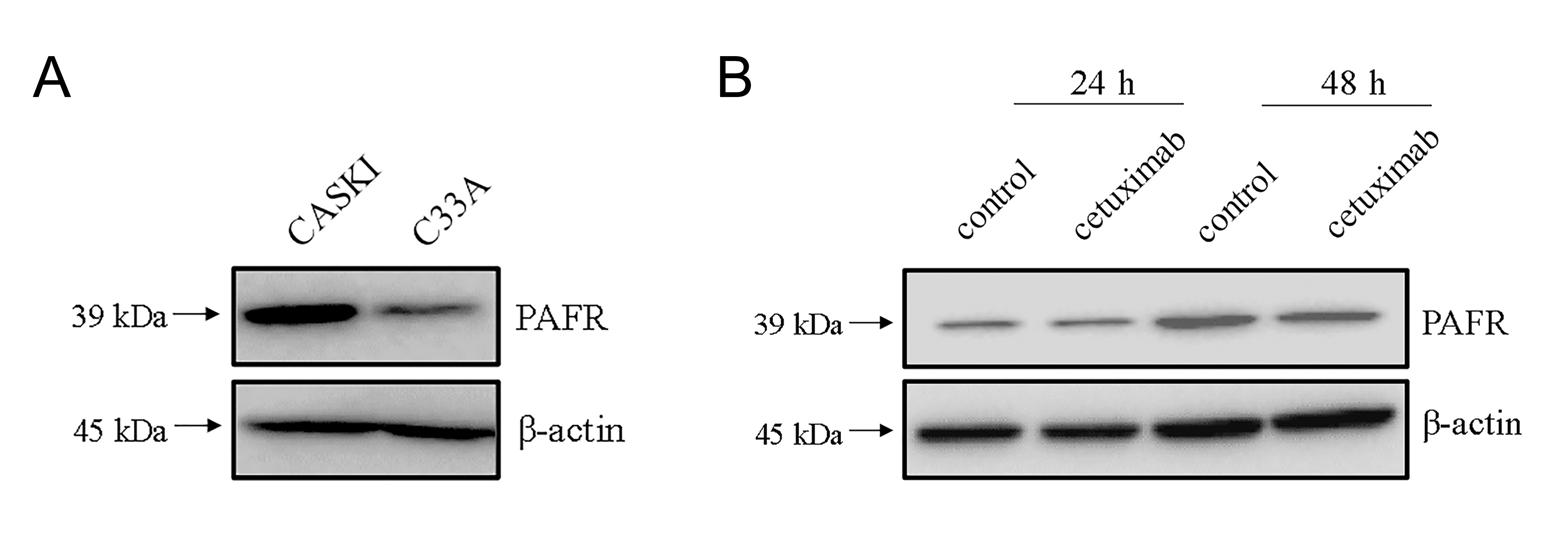

Supplement: Supplementary Figure 4 — PAFR protein levels are differentially expressed in cervical cancer cell lines and regulated by EGFR. (A) CASKI and C33A cell lines were lysed and the levels of PAFR and β-actin (loading control) were determined by Western blotting. Representative image of three experiments. (B) CASKI was treated with cetuximab (100 µg/ml) for 24 h or 48 h in serum-free medium, when cells were lysed and the levels of PAFR and β-actin (loading control) were determined by Western blotting. [file Image_4.tif]
